# Tiger prowling: Distribution modelling for northward-expanding *Aedes albopictus* (Diptera: Culicidae) in Japan

**DOI:** 10.1371/journal.pone.0303137

**Published:** 2024-05-09

**Authors:** Chao Yang, Kyoko Futami, Naoko Nihei, Ryosuke Fujita, Kazumasa Ogino, Kimio Hirabayashi, Mayuko Yonejima, Yasushi Otsuka, Satoshi Nakamura, Kensuke Taira, Makoto Owhashi, Mitsugu Motoki, Tomoyuki Hashimoto, Keiko Minagawa, Shinji Kasai, Yukiko Higa

**Affiliations:** 1 Department of Medical Entomology, National Institute of Infectious Diseases, Shinjuku, Tokyo, Japan; 2 Institute of Tropical Medicine, Nagasaki University, Nagasaki, Nagasaki, Japan; 3 Laboratory of Sanitary Entomology, Faculty of Agriculture, Kyushu University, Fukuoka, Fukuoka, Japan; 4 Department of Immunology and Parasitology, University of Occupational and Environmental Health, Kitakyushu, Fukuoka, Japan; 5 Institution of Textile Science and Technology, Academic Assembly, Shinshu University, Matsumoto, Nagano, Japan; 6 Faculty of Humanities and Social Sciences, Kumamoto University, Kumamoto, Kumamoto, Japan; 7 International Center for Island Studies, Kagoshima University, Kagoshima, Kagoshima, Japan; 8 Faculty of Nursing, Hiroshima Bunka Gakuen University, Kure, Hiroshima, Japan; 9 Laboratory of Parasitology, School of Veterinary Medicine, Azabu University, Sagamihara, Kanagawa, Japan; 10 Tokushima University, Tokushima, Tokushima, Japan; 11 Apex Pest Control Co. Ltd., Minato, Tokyo, Japan; 12 Environmental Biology & Living Environment Department, Japan Environmental Sanitation Center, Kawasaki, Kanagawa, Japan; University of Pécs: Pecsi Tudomanyegyetem, HUNGARY

## Abstract

The Asian tiger mosquito, *Aedes albopictus*, is a significant public health concern owing to its expanding habitat and vector competence. Disease outbreaks attributed to this species have been reported in areas under its invasion, and its northward expansion in Japan has caused concern because of the potential for dengue virus infection in newly populated areas. Accurate prediction of *Ae*. *albopictus* distribution is crucial to prevent the spread of the disease. However, limited studies have focused on the prediction of *Ae*. *albopictus* distribution in Japan. Herein, we used the random forest model, a machine learning approach, to predict the current and potential future habitat ranges of *Ae*. *albopictus* in Japan. The model revealed that these mosquitoes prefer urban areas over forests in Japan on the current map. Under predictions for the future, the species will expand its range to the surrounding areas and eventually reach many areas of northeastern Kanto, Tohoku District, and Hokkaido, with a few variations in different scenarios. However, the affected human population is predicted to decrease owing to the declining birth rate. Anthropogenic and climatic factors contribute to range expansion, and urban size and population have profound impacts. This prediction map can guide responses to the introduction of this species in new areas, advance the spatial knowledge of diseases vectored by it, and mitigate the possible disease burden. To our knowledge, this is the first distribution-modelling prediction for *Ae*. *albopictus* with a focus on Japan.

## Introduction

*Aedes albopictus*, the Asian tiger mosquito, is an invasive species continuously expanding its habitat globally. It originated in the forest fringes of Asia [1] and subsequently spread to nearby islands in the Indian and Pacific Oceans [2] and to European countries and the Americas [3, 4] through historical and contemporaneous trade. The movement of this mosquito via ocean transport has also been confirmed in its native habitat [5]. The invaded areas are primarily located across tropical and temperate regions, with a likely trend of reaching subarctic regions [6]. In addition to its ability to disperse via trade routes, other specific characteristics, e.g. desiccation resistance at the egg stage and a preference for small natural and artificial habitats at immature stages, have helped it overcome harsh conditions during dispersal [1, 7]. Another characteristic of its egg stage, i.e. diapause, allows it to withstand rigorous conditions during winter while entering temperate or subarctic regions, facilitating its establishment in cold areas [8–10]. This species has demonstrated adaptability and resilience to various environments, making it challenging to control its spread. Thus, it has extended its habitat range to all human-inhabited continents [6]. In Japan, its distribution has experienced an unprecedented increase over the past 70 years. It is believed to have migrated from tropical Asia to mainland Japan, although the exact date of this event remains unknown [11] and has existed in Japan for at least 300 years [12]. Before the mosquito survey performed by the United States occupational forces, this species was prevalent in south of Kanto area, including Kyushu, Shikoku, and western Honshu. After its northernmost distribution was determined in the Tochigi Prefecture (~37° N) in 1950 [13], it expanded northward in Japan, where anthropogenic and climatic conditions were favourable. It was first identified in Sendai (~38° N) in 1968. During 1993 and 2000, the northernmost distribution of this species expanded to 39°–40° N [11, 14]. Recently, it was reported to colonise the northernmost part of Honshu Island (~41° N), extending its range (C. Yang, personal communication, sampled in August 2022). As global warming and human transportation continue to affect the environment, it is expected to expand its range to colder regions than previously colonised when relatively more suitable habitats emerge.

*Aedes albopictus* is a competent vector of filariasis and several arboviruses, including dengue and chikungunya viruses, significantly impacting global health [15, 16]. The incidence and intensity of dengue viral infections have increased dramatically over the past 50 years [17], with novel outbreaks reported in invaded regions such as Italy and France [18–20] and repeated outbreaks reported in native areas such as China [21]. Dengue fever is estimated as a threat to half the global population at risk of infection [22]. Several autochthonous dengue outbreaks have been reported in Japan, including in western Japan from 1942 to 1945 and in Tokyo in 2014, which profoundly affected tens of thousands and hundreds of cases, respectively. *Aedes albopictus* is believed to play a crucial role in its transmission, being the only competent vector of the virus in Japan [23, 24]. Considering the potential for further outbreaks due to the spread of this species in northern Japan, the prediction of *Ae*. *albopictus* distribution is required to advance the spatial perception of associated viruses and develop a management strategy for arbovirus control.

Studies have reported the global and local distributions of *Ae*. *albopictus* based on various perspectives [6, 25–31], with two studies specifically focusing on predicting its distribution in Japan [14, 32]. However, a global distribution model of *Ae*. *albopictus* may not accurately reflect its distribution in Japan, as the generated maps tend to overestimate its presence in Japan, including in forest areas in southern Japan unsuitable for mosquitoes. This inaccuracy may be due to the limited occurrence records or the use of environmental variables not suitable for the local area, resulting in a model-fitting bias [31]. In addition, these models use a coarse resolution of 5 km × 5 km and cannot provide detailed information regarding local areas. These studies focused on Japan and used a single variable, temperature, for the prediction [14, 32]. Nawrocki et al. (1987) [32] and Kobayashi et al. (2002) [14] indicated that the minimum mean temperatures of −5 to −2°C during the coldest months and the annual mean temperature of >11°C could ensure *Ae*. *albopictus* survival. Based on these criteria, they constructed a rough distribution map of *Ae*. *albopictus* in Japan rather than refined predictions. In addition to temperature, precipitation and anthropogenic factors affect mosquito establishment [6]. Thus, the actual habitats may have been over- or underestimated. Therefore, rough distribution predictions cannot confirm mosquito distribution and cannot be used to assist public health practitioners in decision-making to prevent arbovirus outbreaks, e.g. dengue fever virus, vectored by mosquitoes.

Considering the crucial role of vectors and the pressing need to improve precision for predicting the distribution of *Ae*. *albopictus*, a comprehensive distribution prediction model is required. We propose a fine-scaled model for predicting the current and future states of *Ae*. *albopictus* in Japan, considering the increasing impacts of climatic and anthropogenic factors. We used a random forest machine-learning model to predict its current and future distributions and estimated the areas and populations at risk of dengue viral infections based on its future distribution. The variable contributions and model evaluations are also discussed.

## Materials and methods

### Mosquito coordinate data

Mosquito presence data were obtained through a nationwide survey conducted in Japan from 2020–2022. Additional data were obtained from the Global Biodiversity Information Facility (http://www.gbif.org) using the ‘dismo’ package in R-4.1.3 on the RStudio platform [33–35] and Yang et al. (2021), who collected data from 2018–2020 [5]. Raw data were cleaned to remove duplicate records and sites lacking coordinates or outside the target boundary. To avoid spatial autocorrelation in modelling, we thinned the data such that each record matched one grain of raster. In total, 523 coordinates were obtained (S1 Table). As *Ae*. *albopictus* is an invasive species, models predicting occupied areas rather than suitable habitats are relatively more appropriate, and using absence data is necessary. As obtaining absence data can be difficult, we used randomly generated 1050 pseudo-absence sites to create a balanced sampling strategy (S1 Table) [36]. Thus, we used 1573 presence and pseudo-absence sites as the dataset for this study (S1 Table). Data cleaning, thinning and pseudo-absence data generation were performed using dismo, sp, raster and spThin packages in R [35, 37–40].

## Environmental variables

Environmental variables affecting the distribution of *Ae*. *albopictus* include elevation, climatic, and anthropogenic factors [6, 25–27, 41]. Elevation data, current and future climate data on temperature and precipitation from the sixth version of the Model for Interdisciplinary Research on Climate, and data on urban areas and population were obtained from WorldClim and Socioeconomic Data and Applications Centre at a resolution of 30 arcs (~1 km × 1 km) [42–46]. The global data were clipped to Japan. The microclimate is essential for insect breeding [27], and relative humidity (RH) is another factor contributing to the habitat conditions required for mosquito breeding. As raster data regarding RH are difficult to obtain, and RH is related to mean and dew point temperatures, we derived them approximately from the following formulae: [47] [48–50]

T−Td=0.0023*h+0.37*T+0.53*R+0.35*Rann−10.9°C, (1)

$$RH=100*\left\{\frac{\exp\left[17.625*\frac{Td}{243.04+Td}\right]}{\exp\left[17.625*\frac{T}{243.04+T}\right]}\right\},$$ (2)

where *T* is the mean temperature, *T*_*d*_ is the dew-point temperature, *h* is the elevation, *R* is the mean daily temperature range, and *R*_*ann*_ is the difference between mean temperatures during the hottest and coldest months. As mean temperatures of the hottest and coldest months in the future were unavailable in WorldClim, we substituted them with mean temperatures of the hottest and coldest quarters. Raster manipulation was performed using dismo and raster packages in R [35, 38].

Twenty-three environmental variables, including bioclimatic variables 1–19, elevation, RH, population, and urban land fraction, were obtained [42–46]; however, not all were used for model fitting owing to collinearity problems and modelling simplicity. Variable selection was based on correlation analysis and the relevance of ecology to this species [25, 26]. The variables considered were not significantly inter-correlated when the Pearson correlation coefficient was <0.8 (S2 Table and S1 Fig), calculated using ENMTools [51]. The variables included mean diurnal range (Bio2), isothermality (Bio3), annual temperature range (Bio7), mean temperature of the wettest quarter (Bio8), mean temperature of the coldest quarter (Bio11), annual precipitation (Bio12), precipitation seasonality (Bio15), precipitation in the coldest quarter (Bio19), annual RH (RH), population (Pop), urban land fraction (Urban), and elevation (Elev; S2–S13 Figs).

Future data on climate, urban development, and demographic changes are dynamic and contingent on societal progression. The ‘shared socioeconomic pathway (SSP)’ narratives, developed by the Intergovernmental Panel on Climate Change [52, 53], were used to backcast societal development. The SSP1 (sustainability) scenario describes a developmental trajectory characterised by increased investment in education and health, resulting in high-income growth and a rapid demographic transition, leading to minimal population growth in high-fertility countries. Contrastingly, a positive outlook on economic prospects maintains moderate fertility in low-fertility countries. Migration and urbanisation are prevalent, with development characterised by rapid urbanisation. The SSP2 (middle of the road) scenario describes a world wherein demographic outcomes align with moderate expectations regarding population growth, urbanisation, and spatial developmental patterns. The SSP3 (regional rivalry) scenario envisions low investments in human capital and low-income growth, leading to high fertility and population growth rates in high-fertility countries. Contrastingly, economic uncertainty leads to low fertility rates and slow population growth (or decline) in low-fertility countries. Rates of migration and urbanisation are low. The SSP5 (fossil-fuelled development) scenario resembles SSP1 but exhibits high fertility in low-fertility countries and poorly managed urbanisation [53]. On top of these, various climatic changes have been predicted to occur before 2100 based on assumed greenhouse gas emission scenarios (SSP126, SSP245, SSP370, and SSP585) with different radiative forcing values (2.6, 4.5, 7.0, and 8.5 W/m^2^, respectively), proposing an increase of 2–5°C in temperature by the end of the century. The urban and population data predictions for the future are also consistent with these SSP narratives [43–46].

### Modelling

For model prediction, we used the random forest machine-learning approach. This method uses multiple decision trees to achieve adequate predictive performance, with each tree grown randomly on an independent bootstrap sample from the training data. Furthermore, the predictor variables at a node inside a tree can be randomly selected [54], further enhancing the prediction capabilities of the model. Considering these factors, the random forest method is an outstanding approach for predicting species distribution. To correlate and predict the relationships between site data and covariate values, we used a random forest model with 1000 trees trained after tuning three predictor variables randomly selected at each iteration (S14 Fig). Sensitivity analysis was conducted to generate a binary map, and variable importance was assessed using the Gini impurity criterion. A low Gini impurity index indicates relatively more accurate pixel classification. Therefore, the mean decrease in Gini impurity index value was calculated for each variable and ranked relative to that of the other variables in the model. This step was followed by the construction of partial dependency plots for each variable to determine the probability of mosquito occurrence. The global behaviour of the model was explained based on the Gini impurity index and partial dependency plot indices, whereas Shapley values were used to interpret the contribution of each variable to local observations [55]. As the Shapley value plot can evaluate the reliability of the model and variable importance at a site level [56], we randomly selected five sites with occurrence possibility from high to low, i.e. 100% to 0% at interquartile intervals, to interpret whether the fitted model was reliable and ecological plausibility. The predictive performance of this model was evaluated using the area under the receiver operating characteristic curve (AUC), calculated as the mean AUC for each of the five cross-validation folds evaluated against the remaining 80% of the data. In addition, we determined the sensitivity, specificity, and true skill statistics of the model. The modelling was executed in R-4.1.3, using the randomForest, rfUtilities and DALEX packages [57–59].

### Estimation of exposed areas and populations

**Fig 1:**
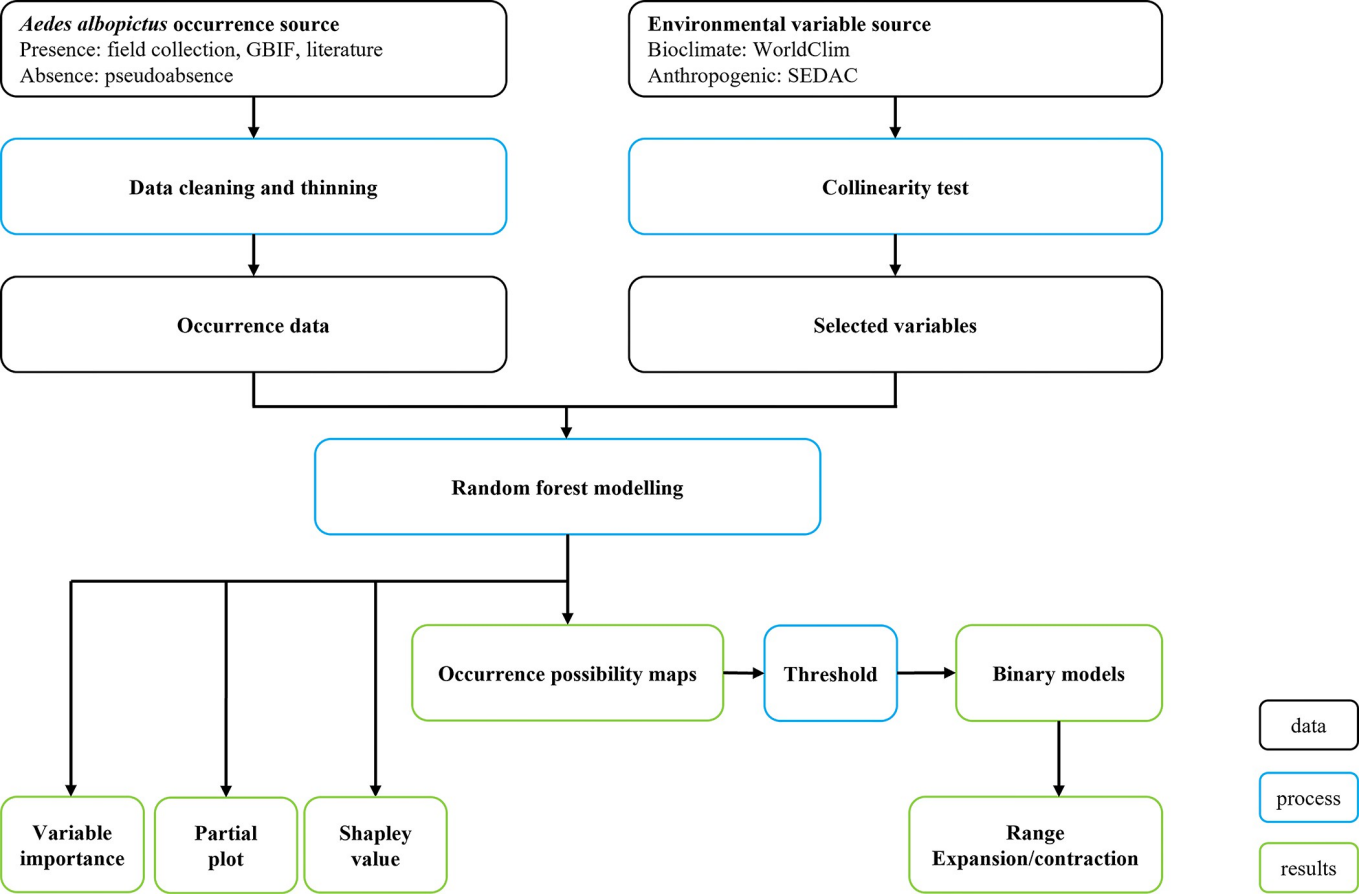
Schematic representation of the study. Black boxes represent data, blue boxes represent analytic processes, and green boxes represent results.

Using a binary model to convert continuous suitability maps into presence/ absence maps, where presence denotes areas at risk, is a commonly employed approach for classifying regions as at-risk or unresponsive to *Ae*. *albopictus* expansion. The value maximising the true-positive and true-negative rates when classifying the occurrence and absence data using the current prediction map was used as the threshold for the binary model. Specifically, a value of 0.35 was determined as the optimal threshold in the random forest model. Subsequently, any pixel exhibiting a predicted occurrence value above this threshold was considered at risk, and the same threshold was applied to each scenario at each time point to calculate the area at risk. The final maps were subsequently overlaid with human population estimates, and the relevant at-risk populations were extracted. Exposed areas and populations were estimated using the raster package in R [38]. A schematic illustration of the study concept and its processes is presented in Fig 1.

## Results

### Current and future distribution ranges and at-risk populations

**Fig 2:**
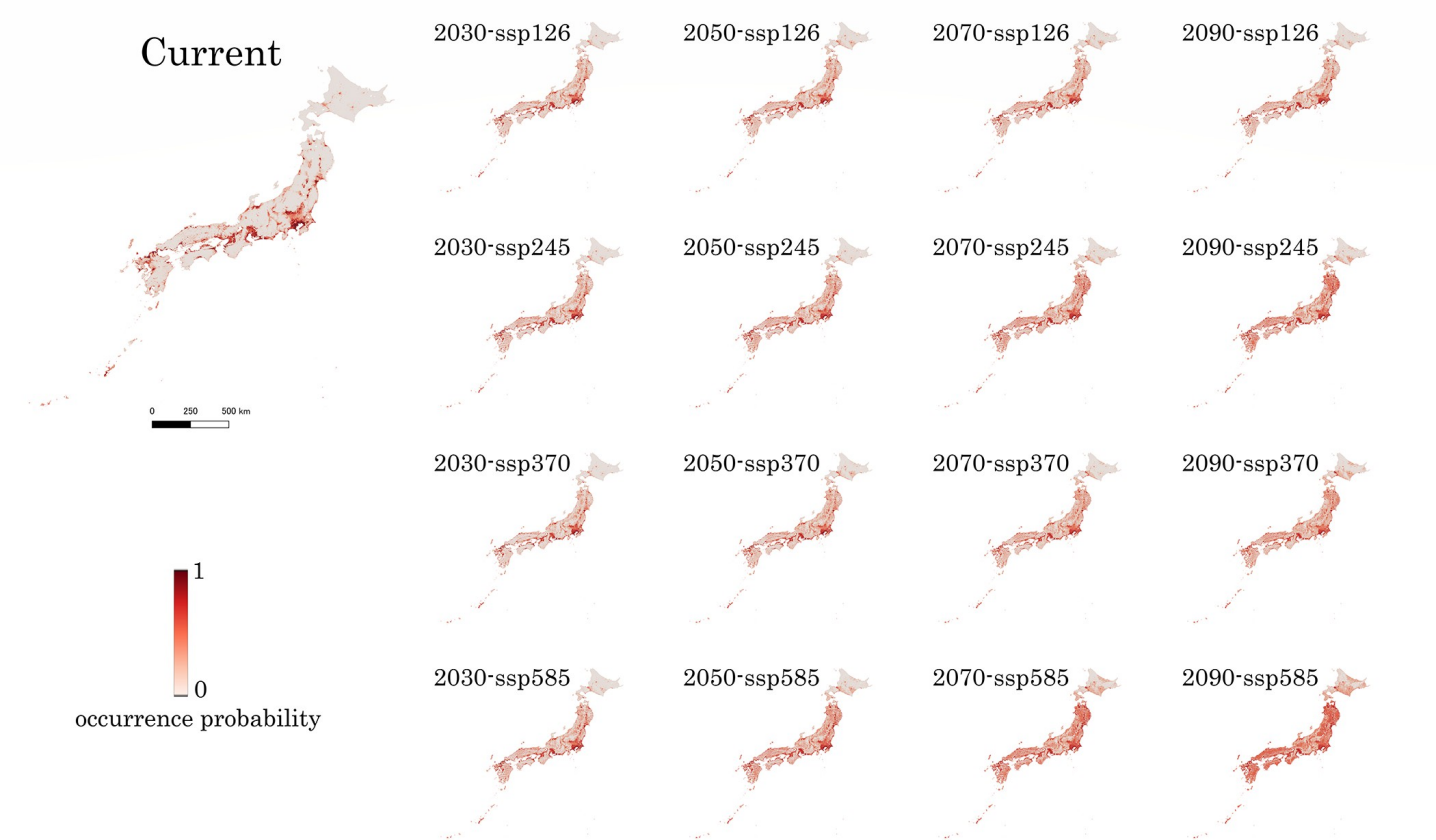
Current distribution prediction for *Aedes albopictus* and various future versions under the four climatic scenarios at four different time points. The deeper the red colour, the higher the probability of occurrence.

**Fig 3:**
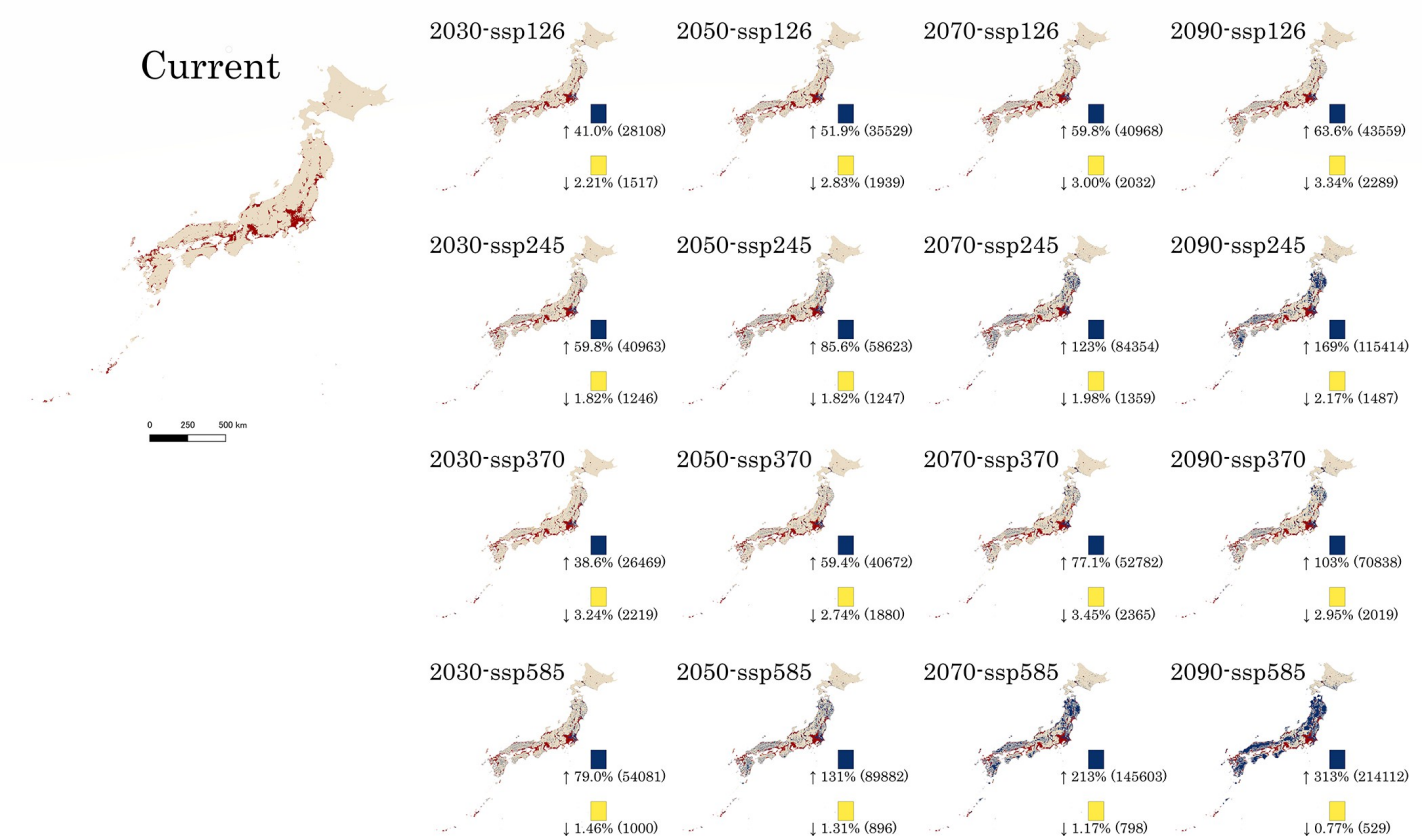
Suitable habitat variation for *Aedes albopictus* from current conditions to future climate scenarios. The binary models were generated using a threshold value maximising the true-positive and true-negative rates obtained in the prediction model. The red colour shows the current suitable habitat defined by the threshold value of 0.35, and the blue and yellow colours show the expanded and contracted regions in future climate scenarios, respectively. Percentages and areas (km^2^) of the expanded and contracted regions are shown under each colour bar in each model.

The predicted distributions of *Ae*. *albopictus* in Japan showed that almost all urban areas are occupied by this mosquito (Figs 2 and 3). Kanto, Kansai, Tokai, the Seto Inland Sea, and western Kyushu represented the most habitable areas. Other suitable habitats were located patchily in small cities along the Pacific Ocean, Sea of Japan, in Tohoku District, and Hokkaido. Most areas of Japan are covered by primary and secondary forests, with few records of *Ae*. *albopictus*. This prediction was also supported by observations in uninhabitable areas (Figs 2 and 3, S15 Fig).

In future projections, the situation will vary for each scenario (Figs 2 and 3). With increasing greenhouse gas emissions under the four different scenarios, the temperature would exceed the current predicted range of 2–5°C, and the climatic covariates used here would also vary between the current and future conditions (S2–S10 Figs). In addition, the urban extent increased in all scenarios, whereas the population contracted, except for scenario SSP585 (S11 and S12 Figs). We predicted a significant increase in *Ae*. *albopictus* between 2030 and 2090 (Figs 2 and 3). Specifically, *Ae*. *albopictus* is expected to be established in northeastern Kanto, many areas of Tohoku District, and Hokkaido by 2030 (Figs 2 and 3). In scenario SSP126, the northeastern Kanto areas would be suitable for mosquito breeding, with a trend expanding to the Ibaraki Prefecture, whereas in the remaining regions in Japan, mosquitoes would expand their range from urban to surrounding areas. Towards the end of this century, this range would have contracted slightly in Hokkaido (Figs 2 and 3). In scenarios SSP245 and SSP370, the trend would be the same as in scenario SSP126, with a larger range than previously predicted but minimal contraction. More areas will become suitable habitats in the Tohoku District and Hokkaido. The difference between these two scenarios is the higher urban ratio and population in scenario SSP245 but relatively less favourable climatic conditions than in scenario SSP370 (S2–S12 Figs). Therefore, the habitable range in Honshu is larger in scenario SSP245 than in scenario SSP370; however, the habitable range is reversed in Hokkaido (Figs 2 and 3). In scenario SSP585, which depicted the most severe circumstances resulting from climate change and anthropogenic activity, the species covered the entire land area of Japan, excluding the central highlands and northern and eastern Hokkaido (Figs 2 and 3, S13 Fig).

**Fig 4:**
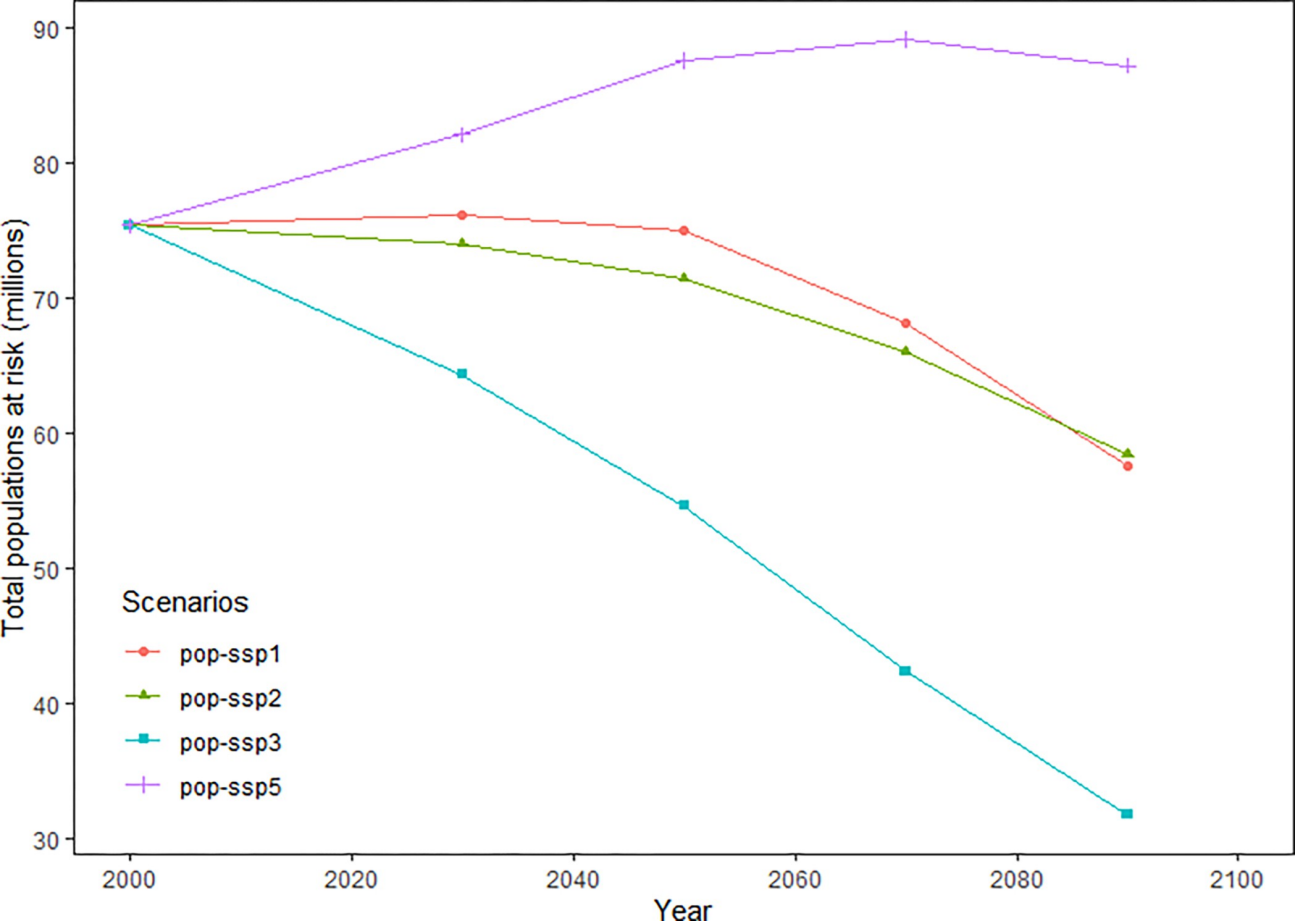
Estimates of exposed population within the predicted distribution range of *Aedes albopictus* under future climate scenarios.

Based on the future distribution predictions, several regions may be suitable for *Ae*. *albopictus* breeding; however, a few areas may have been less suitable for habitation. The expansion areas in the four scenarios at the four different time points increased the least by 38.6% in scenario SSP370 in 2030 and the most by 313% in scenario SSP585 in 2090. Conversely, the contracted areas would decrease by a minimum of 0.77% in SSP585 in 2090 and by 3.45% in SSP370 in 2030 (Fig 3). Furthermore, populations at risk decreased in the previous three scenarios but increased in scenario SSP585, as population projections predicted a demographic decline in scenarios SSP126, 245, and 370 for Japan (Fig 4).

### Predictor contributions and model evaluation

**Fig 5:**
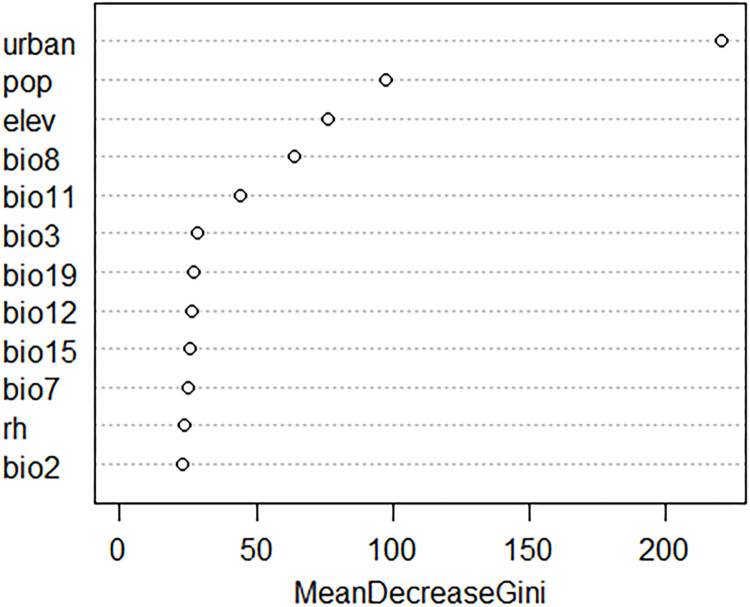
Variable importance of model predictions for *Aedes albopictus* occurrence. Bio2: mean diurnal range, Bio3: isothermality, Bio7: annual temperature range, Bio8: mean temperature of the wettest quarter, Bio11: mean temperature of the coldest quarter, Bio12: annual precipitation, Bio15: precipitation seasonality, Bio19: precipitation in the coldest quarter, RH: annual relative humidity, Pop: population, Urban: urban land fraction and Elev: elevation.

**Fig 6:**
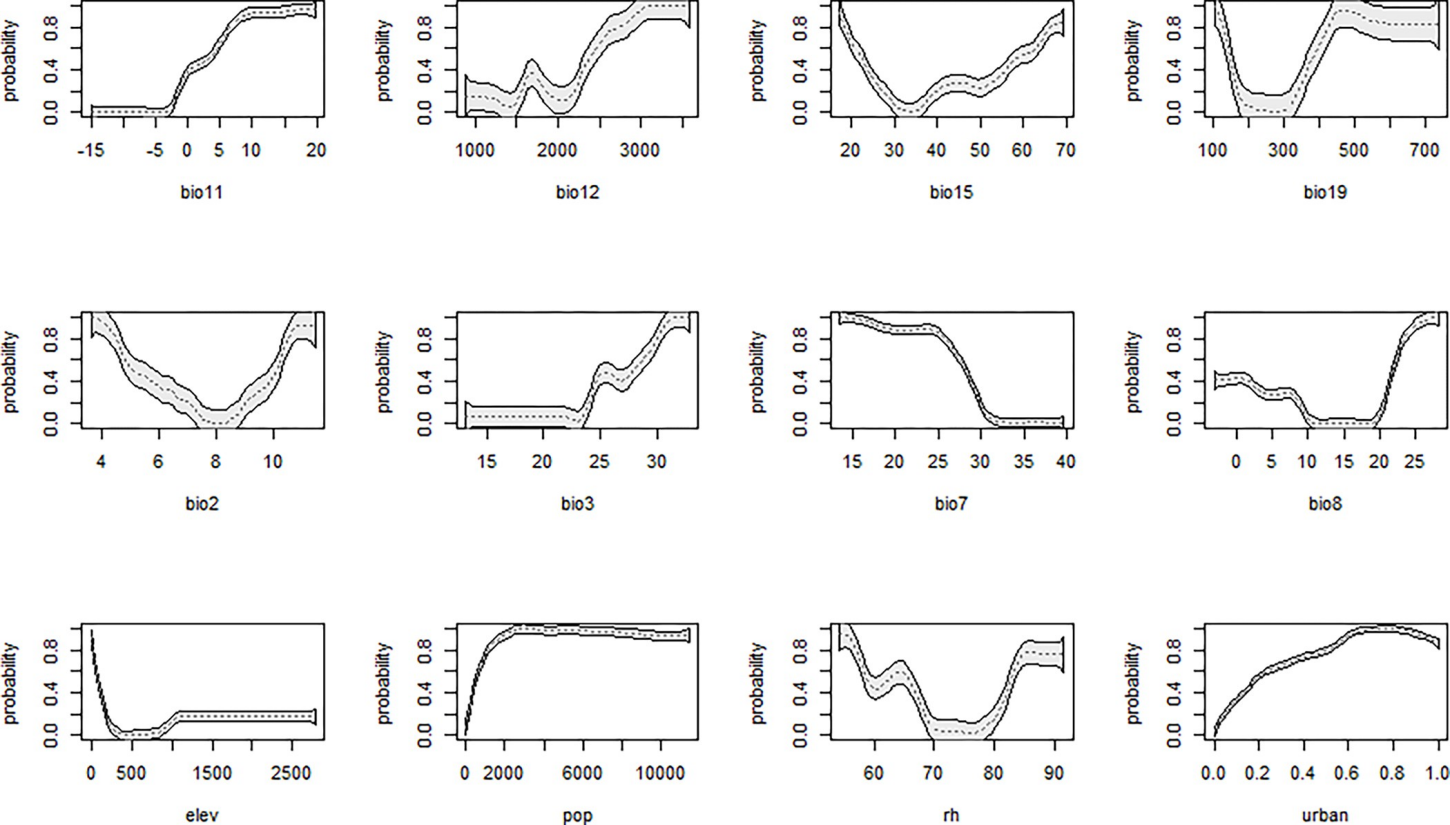
Partial plot for each covariate. The vertical axis represents the occurrence probability of mosquitoes, and the horizontal axis represents the variation range of the related variables. The shaded area represents a 95% confidence interval, and the dashed lines represent the average values. Bio2: mean diurnal range, Bio3: isothermality, Bio7: annual temperature range, Bio8: mean temperature of the wettest quarter, Bio11: mean temperature of the coldest quarter, Bio12: annual precipitation, Bio15: precipitation seasonality, Bio19: precipitation in the coldest quarter, RH: annual relative humidity, Pop: population, Urban: urban land fraction and Elev: elevation.

**Fig 7:**
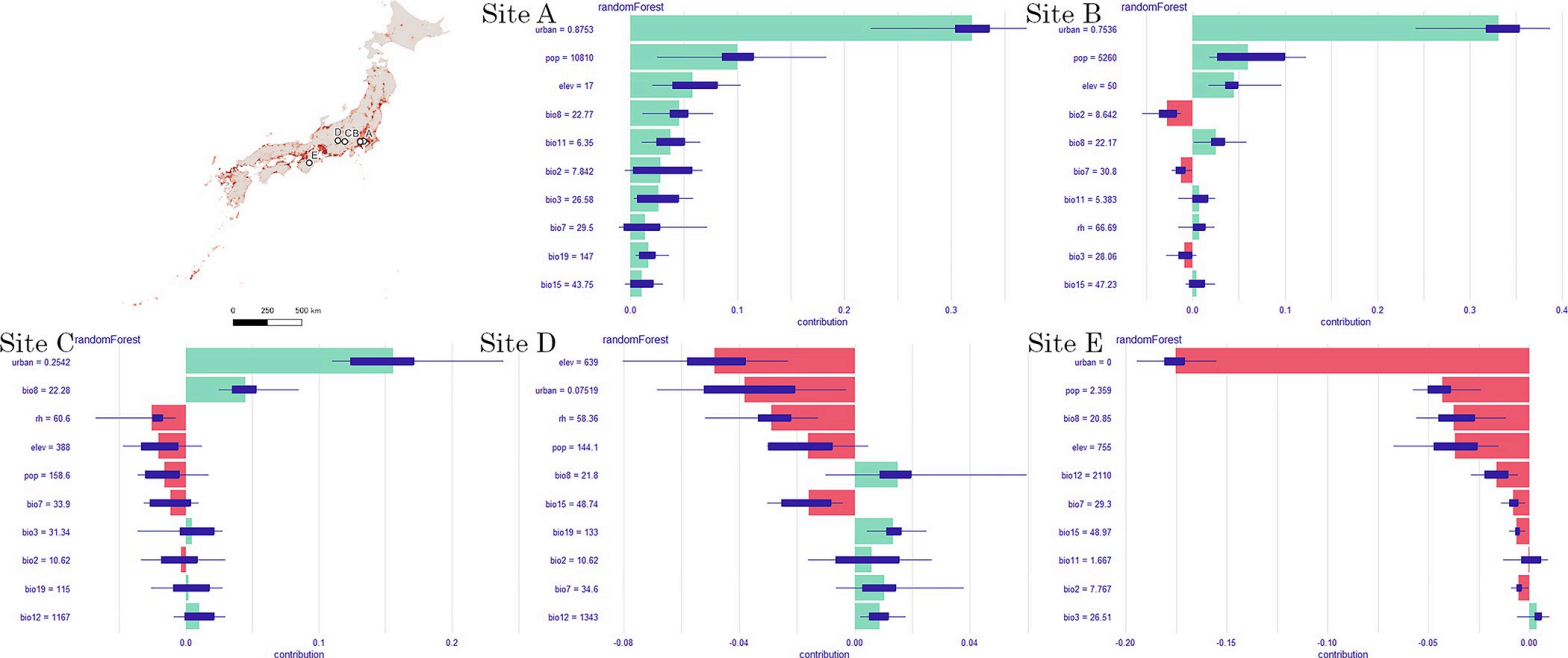
Shapley values plots for observations based on *Aedes albopictus* distribution prediction. The green and red bars correspond to the positive and negative contribution of the variable to the prediction. Purple boxplots display variable attribution distribution with all variable layout combinations. The *y*-axis shows the variables and their observation values, and the *x*-axis shows model prediction values. Observation values for each site: Site A (100%), Site B (75%), Site C (50%), Site D (25%), Site E (0%). Bio2: mean diurnal range, Bio3: isothermality, Bio7: annual temperature range, Bio8: mean temperature of the wettest quarter, Bio11: mean temperature of the coldest quarter, Bio12: annual precipitation, Bio15: precipitation seasonality, Bio19: precipitation in the coldest quarter, RH: annual relative humidity, Pop: population, Urban: urban land fraction and Elev: elevation.

Gini coefficients were used to represent the importance of the predictors in the model, and a partial dependency plot was used to illustrate the relationship between the occurrence probability and range of each covariate when the others remained constant at their mean values. Shapley value plots were used to explain the relative contribution of each feature to the prediction at a given instance. Therefore, the most contributing predictor was the urban land fraction, preceding population, and elevation (Fig 5). Urban areas and population had a positive effect on the occurrence of *Ae*. *albopictus*, whereas elevation had a negative effect (Fig 6). Summer and winter temperatures had less effects on mosquito distribution compared with those of anthropogenic factors (Fig 5). Precipitation and related climatic factor predictors made fewer significant contributions (Fig 5). These relationships are illustrated in Fig 6. When zooming in on the sites that predicted varied occurrence possibilities, the Shapley value plot showed that sites with high urban land fraction, high population, and low elevation were positively associated with high occurrence possibilities, like sites A and B, and vice versa at the sites with low occurrence possibilities, like sites C, D, and E. These results were quite aligned with the global interpretations. It also indicated that the impact of climatic factors was less contributed to the model and had a diverse association at each site (Fig 7). The model predicts the distribution of *Ae*. *albopictus*, with a mean AUC value of 0.94, averaging the 5-fold cross-validation (S16 Fig). A summary of the performance of this model in terms of sensitivity, specificity, and true skill statistics is provided in Table 1.

**Table 1:** Metrics for validation of the model outputs.

Variable	AUC	TSS	Sensitivity	Specificity
Coefficient	0.9482 ± 0.0016	0.5375 ± 0.0051	0.7351 ± 0.0017	0.8024 ± 0.0034

Abbreviations: AUC, area under the receiver operating characteristic curve; TSS, true skill statistics.

## Discussion

Considering the relatively critical ecological and physiological covariates for *Ae*. *albopictus* expansion, we created a set of high-resolution probability maps for its prediction, which will be useful in future studies. Our predictions closely matched the survey data (S15 Fig) and those of previous studies [60–62]. These studies indicate that this mosquito is more prevalent in urban areas than in forested areas in Japan, which aligns with our predictions. The unique topography and population distribution may have affected the availability and preference of blood sources for *Ae*. *albopictus* in Japan. Urban areas in the lowlands may attract species that readily feed on humans, whereas forested regions in the mountains may not provide attractive human blood sources and may be difficult to access [63]. Urban environments provide relatively more habitats for larvae, which directly increases mosquito survival [64]. Therefore, our prediction outperforms previous temperature-only predictions conducted in Japan as it captures relatively more informative data. Earlier studies had limitations in the detection of crucial information [14, 32]. Our predictions were made on a scale of 1 km × 1 km and are relatively more accurate than global predictions on a scale of 5 km × 5 km [6, 27] because the home range of *Ae*. *albopictus* is estimated to be no more than 1 km [65].

These predictions will aid in understanding the spatial distribution of *Ae*. *albopictus* and the arboviruses vectored by it in Japan. Considering the current and future vector distributions, surveillance of the vector and its associated viruses will be relatively more effective. These maps can assist in identifying areas where this mosquito could survive but has not yet been found and selecting the most viable regions for surveillance previously overlooked. For example, the northeastern region of Kanto is projected to be inhabited by *Ae*. *albopictus* by the 2030s, and the Tohoku District is predicted to be mainly inhabited by mosquitoes in the future (Figs 2 and 3).

Both anthropogenic and climatic factors considerably influence the distribution of *Ae*. *albopictus*, with urban areas and populations exhibiting the greatest effects (Figs 5–7). Previous studies have shown that *Ae*. *albopictus* prefers blood meals and urban environments [63, 64], and the possibility of the occurrence of mosquitoes was predicted to increase with urban land fraction and population (Fig 7). The anthropogenic environment is considered conducive to breeding and is a key factor in the establishment of this species during its distribution expansion. Previous studies have supported the importance of urban areas and populations [26, 28, 30, 31]. Summer and winter temperatures were attributed to the proliferation and survival, respectively, of *Ae*. *albopictus*. As mentioned in previous studies [25, 26, 29, 30], these factors limit the broad range of mosquito habitats, i.e. the preferred temperature range for mosquitoes is 20°C to 30°C in summer and not less than 5°C in winter (Fig 7). Despite being less important in global and local assessments, the below variables are essential for mosquito life cycles. The species prefers small water-filled containers at immature stages; therefore, annual precipitation is another factor influencing model prediction, which has been previously validated [25, 30, 31]. Winter precipitation, represented by snow in northern Japan, may also assist in the overwintering of eggs by protecting them from cold temperatures, as reported in a study in the Northeastern United States [26]. In contrast to the results reported by Kraemer et al. (2019) [27], RH had a minimal effect on the distribution at 1 km × 1 km in this study, likely because Japan is surrounded by sea and has a suitable range of moisture nationwide for the species (S10 Fig) or scale selected.

However, the relative contributions of each variable became apparent once the situation changed dramatically in the different scenarios. The strength of the climatic factors became prominent when the anthropogenic factors weakened, as depicted in scenarios SSP245 and SSP370 (Figs 2 and 3). The population and urbanisation ratios increased at a higher rate in scenario SSP245, in contrast to the relatively low rate of increase in scenario SSP370 (S11 and S12 Figs). The range of *Ae*. *albopictus* was marginally larger under scenario SSP245 than under scenario SSP370, except in Hokkaido, where the range was reversed to a great extent under scenario SSP370. Warmer climatic conditions in Hokkaido under scenario SSP370 than under scenario SSP245 (S2–S10 Figs) led to a reversal of the range of *Ae*. *albopictus*. Therefore, interventions for reducing greenhouse gas emissions and anthropogenic activities are critical for shaping the future developmental trends of *Ae*. *albopictus* expansion.

Under future global warming scenarios, *Ae*. *albopictus* is projected to increase significantly under the four greenhouse gas emission scenarios and poses a risk to human health in areas where it can survive and reproduce in Japan. Uncontrolled urban expansion may have contributed to providing suitable habitats for this species. The limitless exploitation of fossil fuels, leading to increased temperatures, favours mosquito habitat expansion. The recovery of Tohoku District from the earthquake disaster in 2011 and bullet train development in Hokkaido likely facilitated its introduction to these areas owing to frequent human movement and domestic trade. Policymakers should plan urban development, limit greenhouse gas emissions, and strengthen entomological surveillance around high-risk introduction routes, e.g. ports and highways in Tohoku District and Hokkaido, and develop protocols for vector control to prevent introduction from established populations. These efforts are expected to intensify as human populations become increasingly connected and urban agglomerations expand.

## Conclusion

This study presented a set of high-resolution distribution maps of the current and future habitation ranges of *Ae*. *albopictus*, the main vector of the dengue virus, in Japan. The distribution of this mosquito species has expanded to major urban areas in Japan. Under the projected environmental changes, its range is expected to expand further, encompassing broader areas of urban habitats and reaching multiple regions in northeastern Kanto, Tohoku District, and Hokkaido. Urbanisation and host availability, expressed as population, facilitate expansion, whereas elevation poses a barrier to its dispersal. Although temperature and precipitation are deemed of secondary importance, they are related to the expansion of the mosquito range. These maps are necessary for public health practitioners to conduct the surveillance of mosquito vectors and associated diseases to increase awareness and preparedness for *Aedes*-borne diseases. To our knowledge, this is the first distribution modelling prediction focused on *Ae*. *albopictus* in Japan.

## Supporting information

S1 Fig Cluster dendrogram of the variables grouped based on the results of the multi-collinearity test. (TIFF)

S2 Fig Spatial distribution (A) and counts (B) of each value for mean diurnal range (Bio2) covariate.
(TIFF)

S3 Fig Spatial distribution (A) and counts (B) of each value for isothermality (Bio3) covariate.
(TIFF)

S4 Fig Spatial distribution (A) and counts (B) of each value for annual temperature range (Bio7) covariate.
(TIFF)

S5 Fig Spatial distribution (A) and counts (B) of each value for mean temperature of the wettest quarter (Bio8) covariate.
(TIFF)

S6 Fig Spatial distribution (A) and counts (B) of each value for mean temperature of the coldest quarter (Bio11) covariate.
(TIFF)

S7 Fig Spatial distribution (A) and counts (B) of each value for annual precipitation (Bio12) covariate.
(TIFF)

S8 Fig Spatial distribution (A) and counts (B) of each value for precipitation seasonality (Bio15) covariate.
(TIFF)

S9 Fig Spatial distribution (A) and counts (B) of each value for precipitation in the coldest quarter (Bio19) covariate.
(TIFF)

S10 Fig Spatial distribution (A) and counts (B) of each value for annual relative humidity (RH) covariate.
(TIFF)

S11 Fig Spatial distribution (A) and counts (B) of each value for population (Pop) covariate.
(TIFF)

S12 Fig Spatial distribution (A) and counts (B) of each value for urban land fraction (Urban) covariate.
(TIFF)

S13 Fig Spatial distribution (A) and counts (B) of each value for elevation (Elev) covariate.
(TIFF)

S14 Fig The best number of predictor variables to fit the model at each iteration. The lower the OOB error, the better the chosen number of variables.
(TIFF)

S15 Fig Current prediction with occurrence data. Blue dots represent presence data, and black dots represent (pseudo-)absence data.
(TIFF)

S16 Fig Evaluation of model performance by area under the receiver operating curve (AUC) ranging from 0–1. The higher the AUC value, the better the predictive performance.
(TIFF)

S1 Table Description of mosquito coordinates used for random forest model prediction. (CSV)

S2 Table Correlation test regarding the collinearity issue of the model. (XLSX)
