# Supplementary figures and images for: Tiger prowling: Distribution modelling for northward-expanding Aedes albopictus (Diptera: Culicidae) in Japan
Source: PLoS One. 2024 May 9;19(5):e0303137. doi: 10.1371/journal.pone.0303137 (PMC11081387; doi:10.1371/journal.pone.0303137)

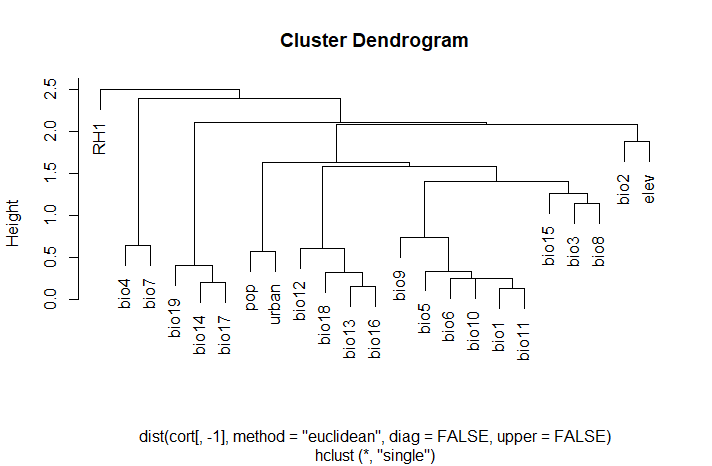

Supplement: S1 Fig — (TIFF) [file pone.0303137.s001.tiff]

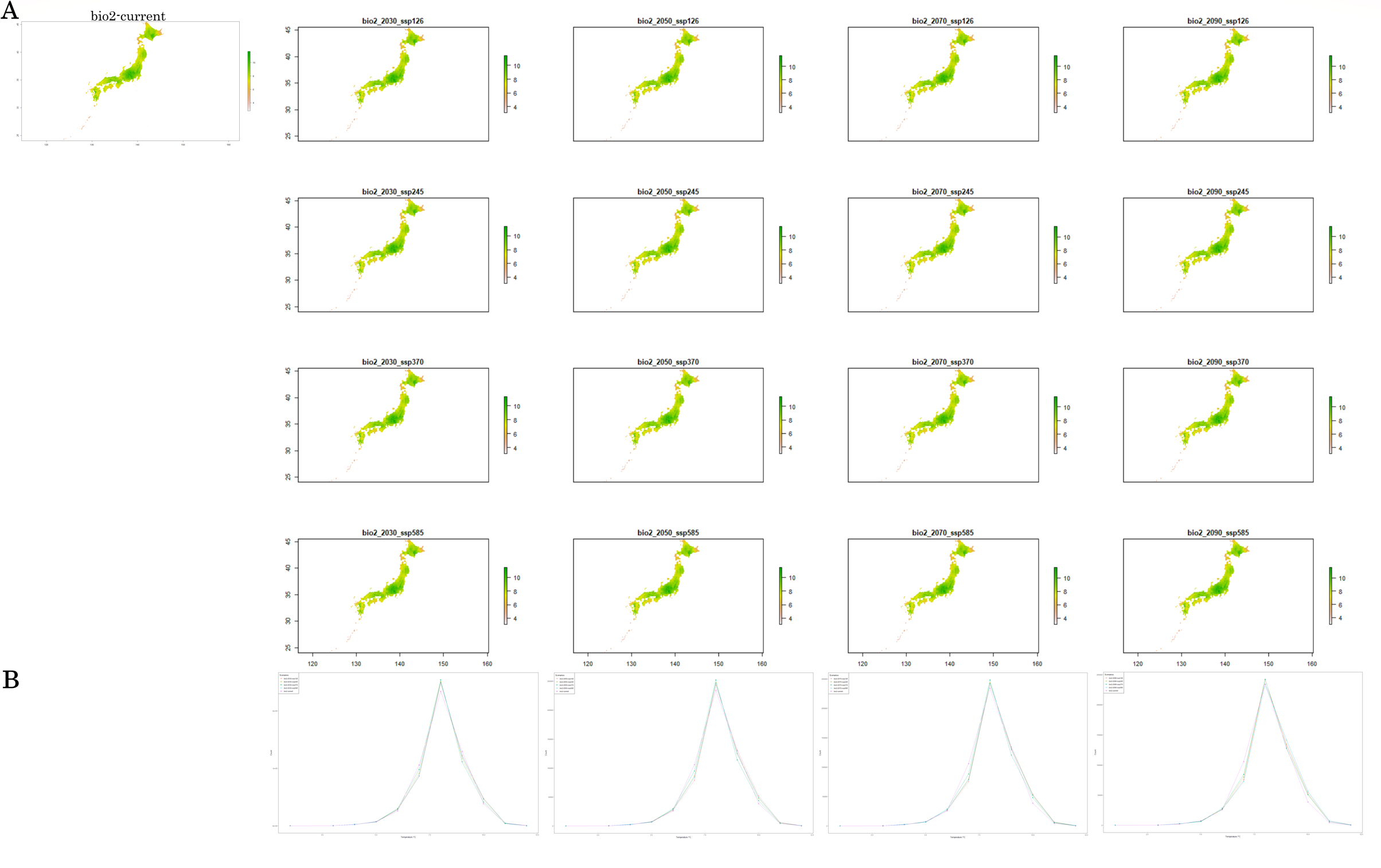

Supplement: S2 Fig — Spatial distribution (A) and counts (B) of each value for mean diurnal range (Bio2) covariate. (TIFF) [file pone.0303137.s002.tiff]

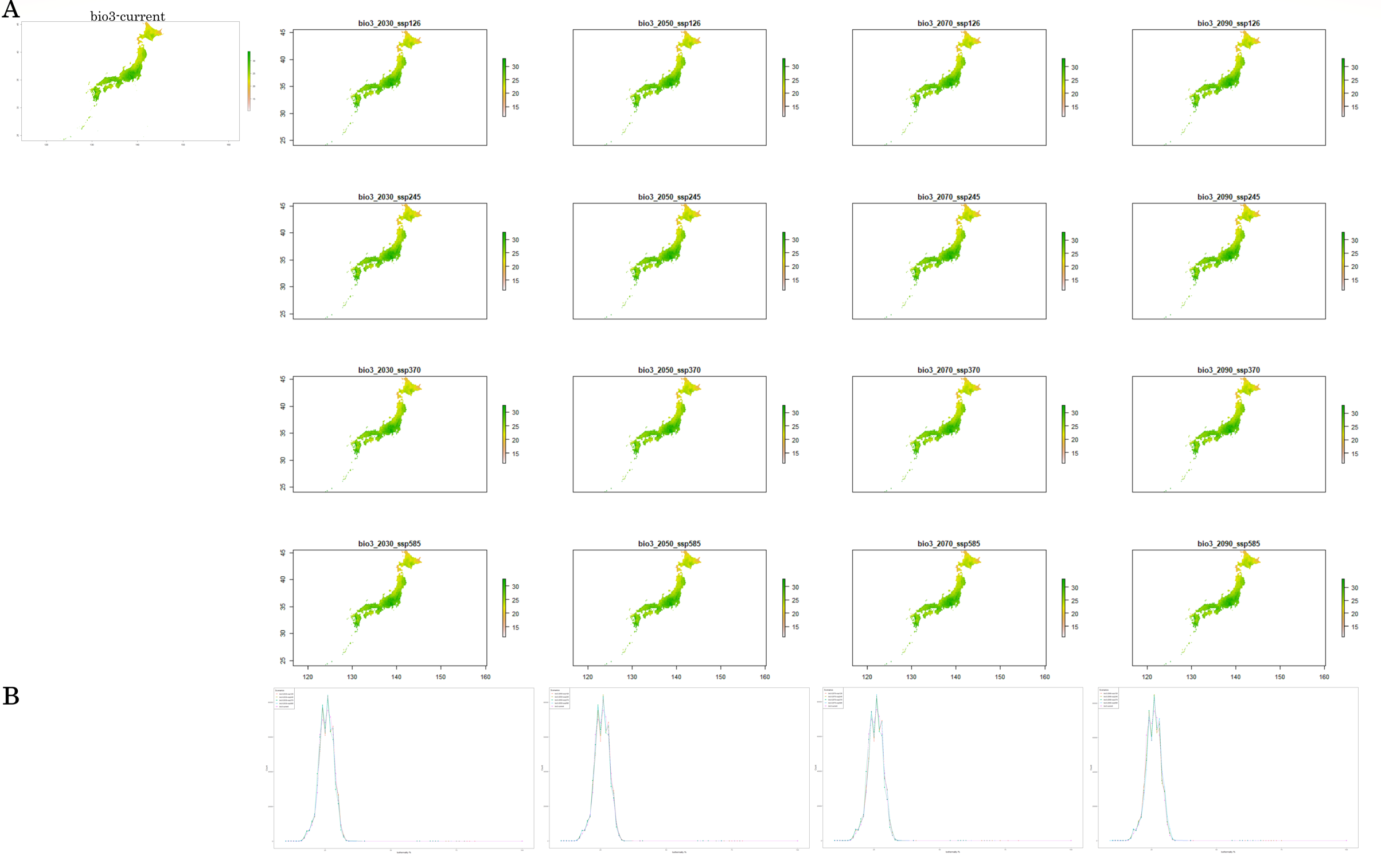

Supplement: S3 Fig — Spatial distribution (A) and counts (B) of each value for isothermality (Bio3) covariate. (TIFF) [file pone.0303137.s003.tiff]

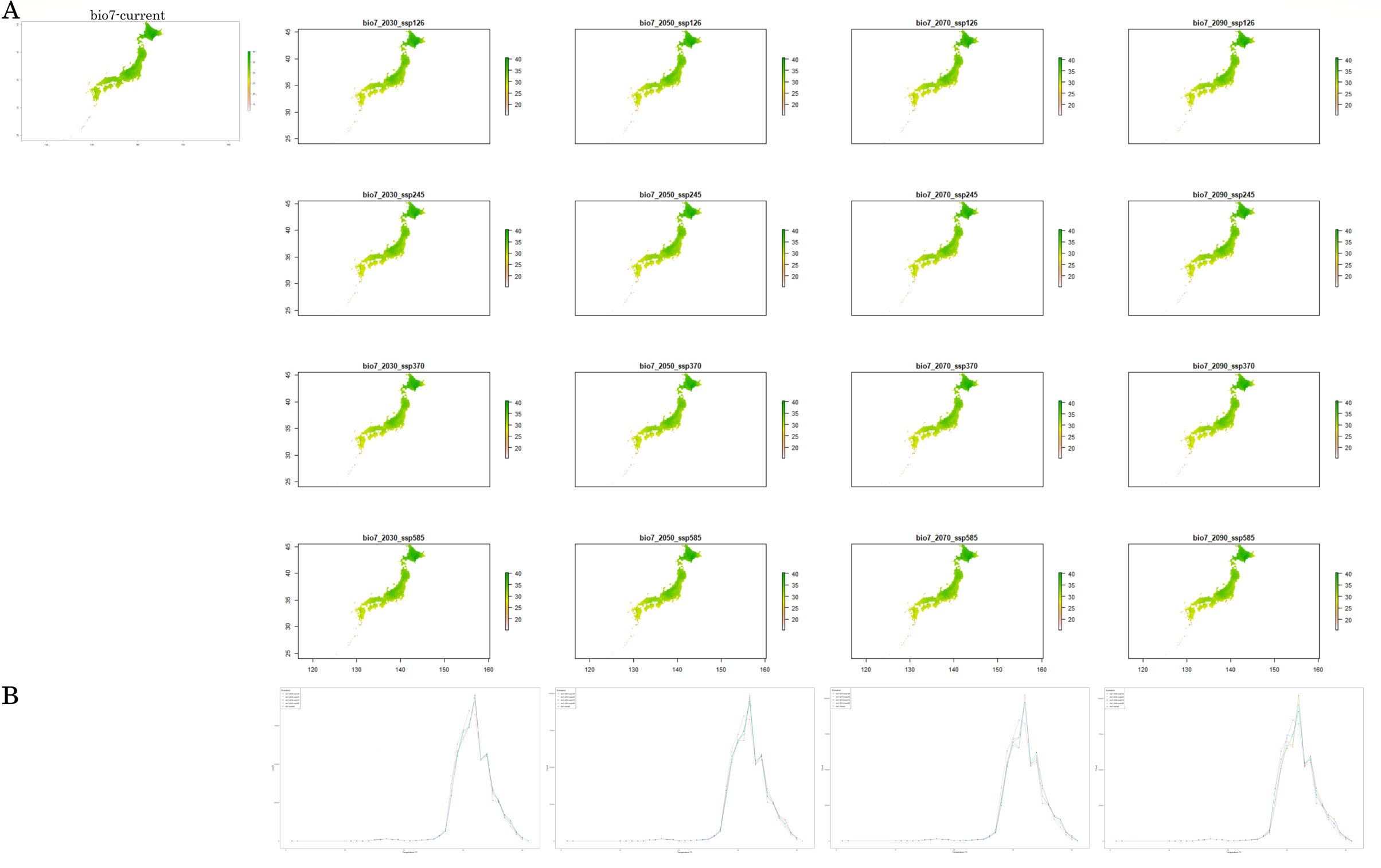

Supplement: S4 Fig — Spatial distribution (A) and counts (B) of each value for annual temperature range (Bio7) covariate. (TIFF) [file pone.0303137.s004.tiff]

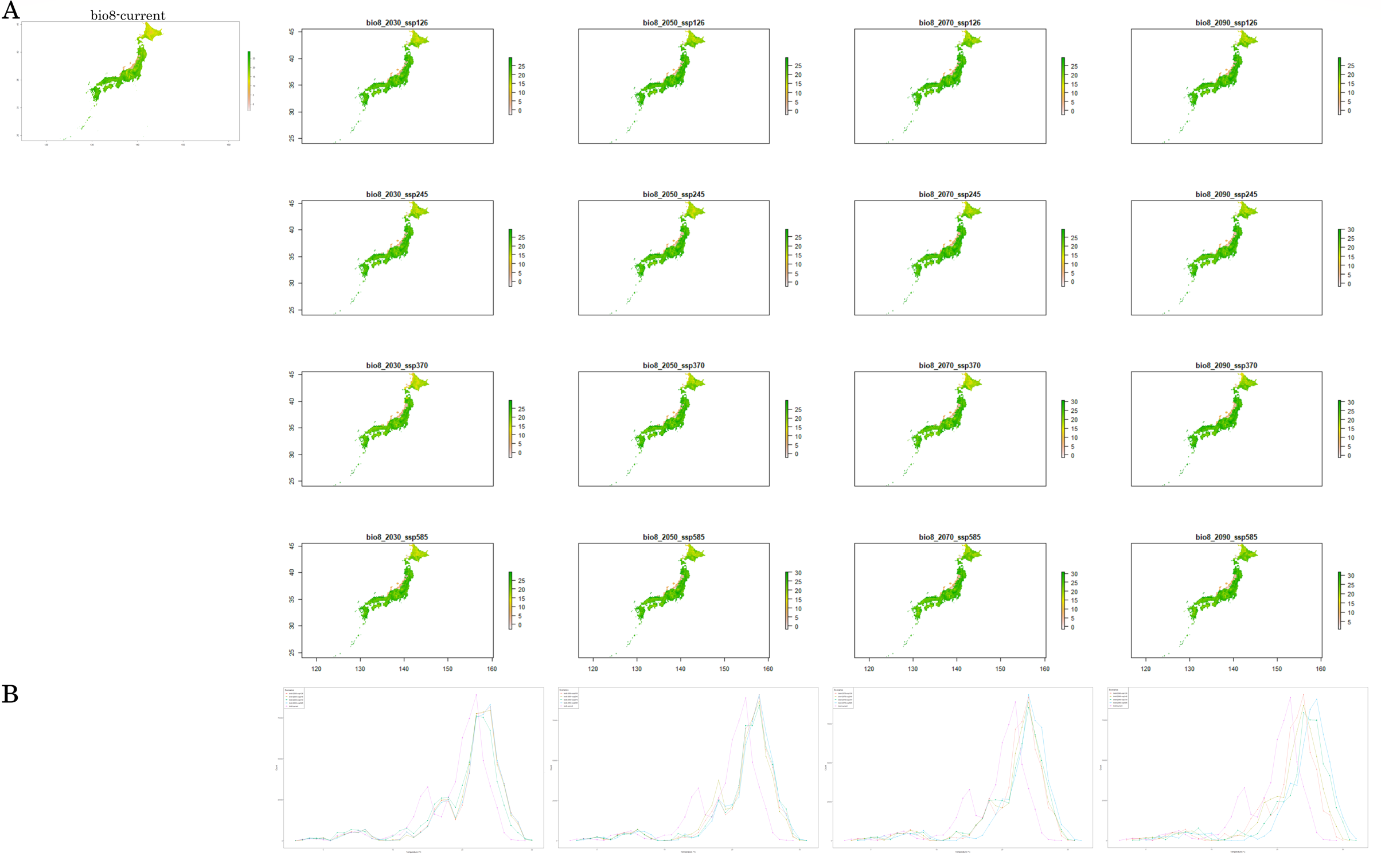

Supplement: S5 Fig — Spatial distribution (A) and counts (B) of each value for mean temperature of the wettest quarter (Bio8) covariate. (TIFF) [file pone.0303137.s005.tiff]

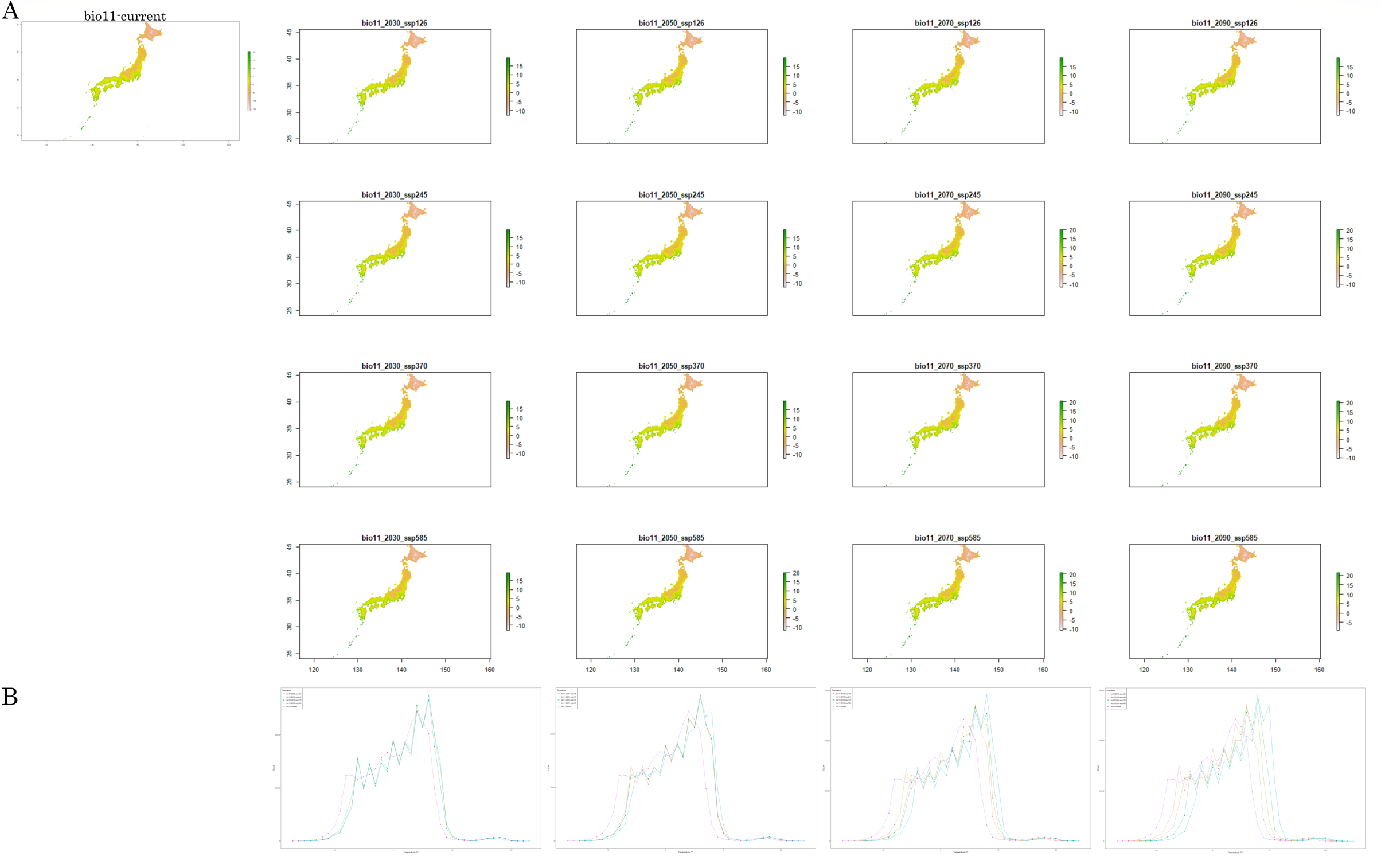

Supplement: S6 Fig — Spatial distribution (A) and counts (B) of each value for mean temperature of the coldest quarter (Bio11) covariate. (TIFF) [file pone.0303137.s006.tiff]

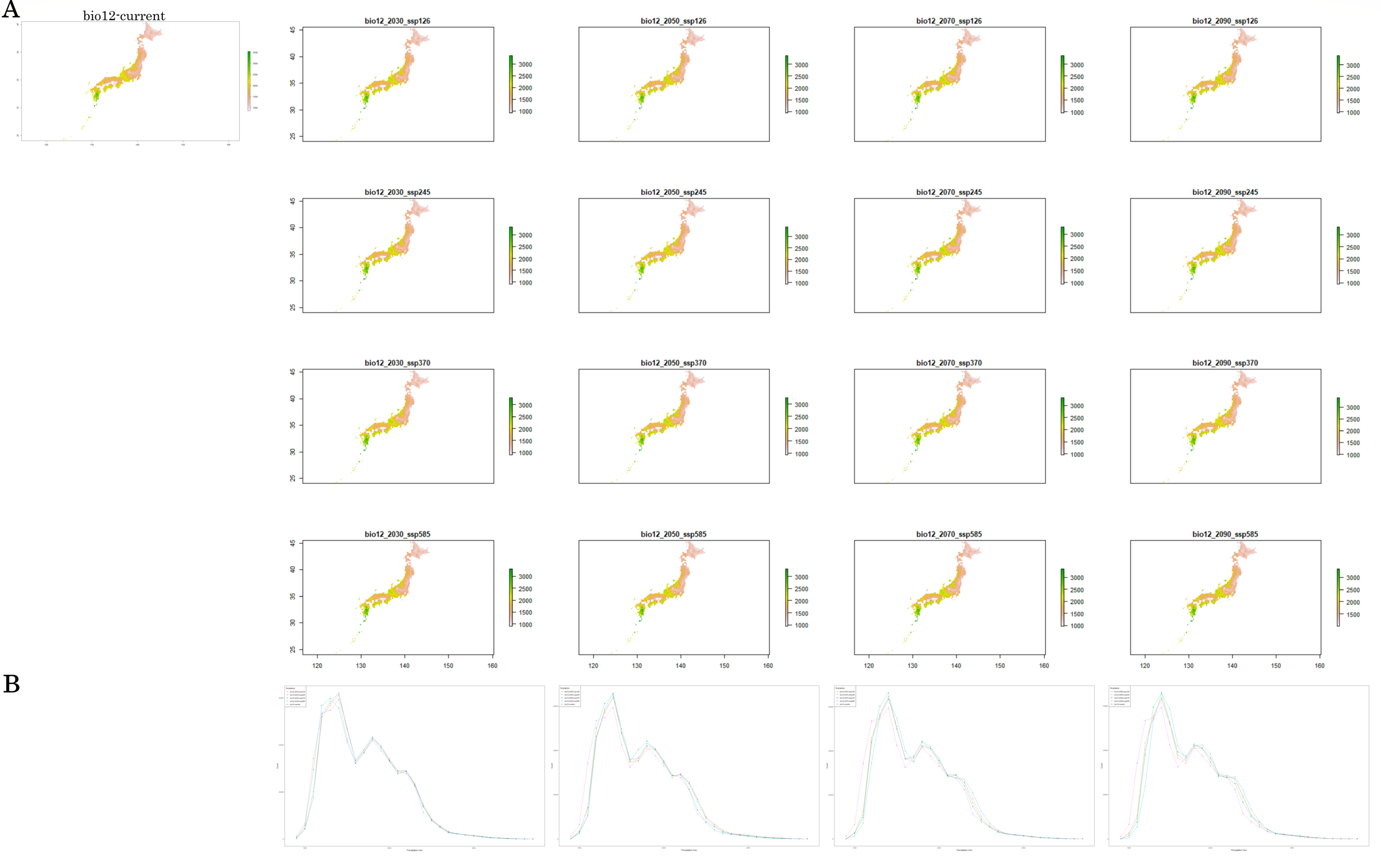

Supplement: S7 Fig — Spatial distribution (A) and counts (B) of each value for annual precipitation (Bio12) covariate. (TIFF) [file pone.0303137.s007.tiff]

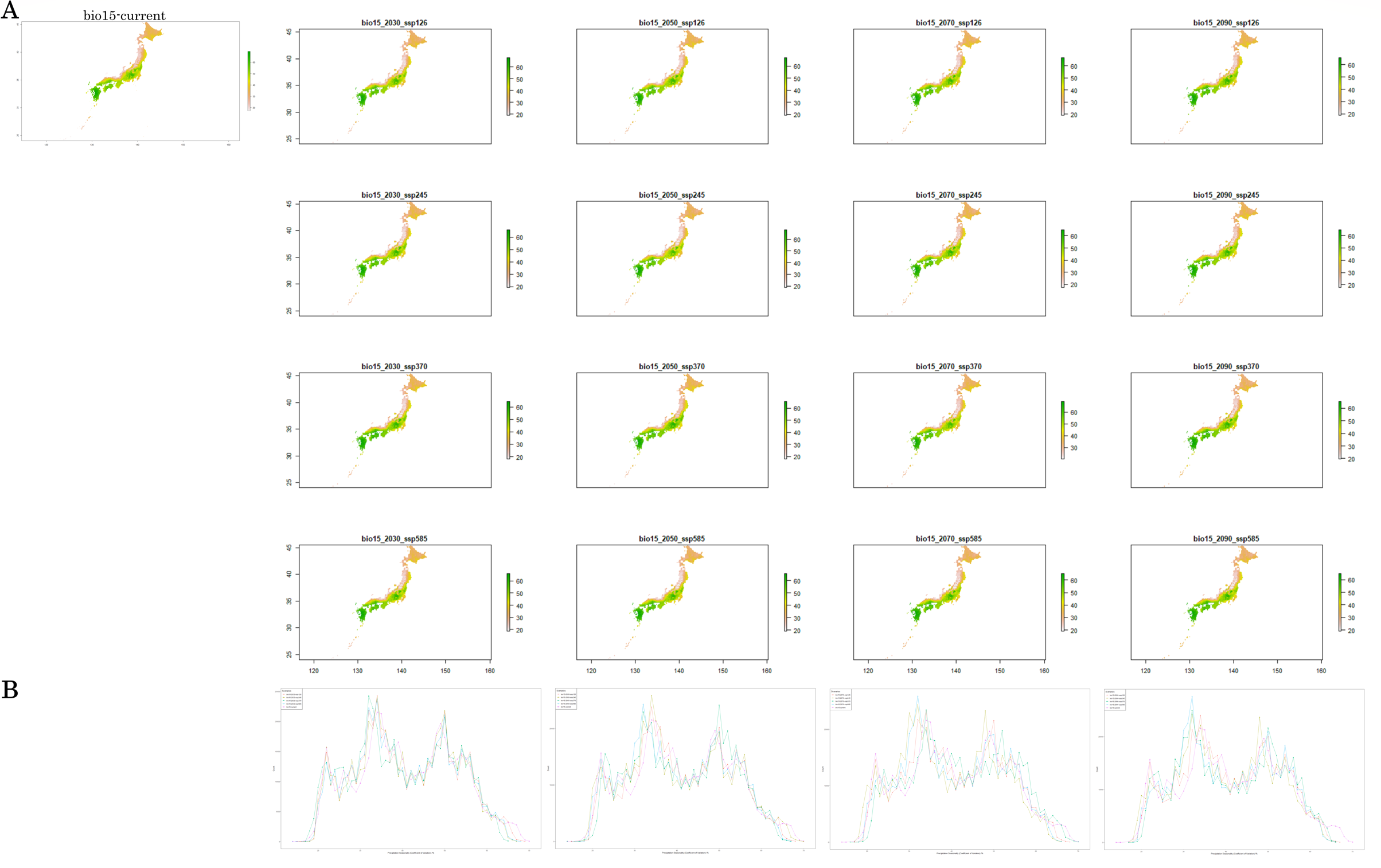

Supplement: S8 Fig — Spatial distribution (A) and counts (B) of each value for precipitation seasonality (Bio15) covariate. (TIFF) [file pone.0303137.s008.tiff]

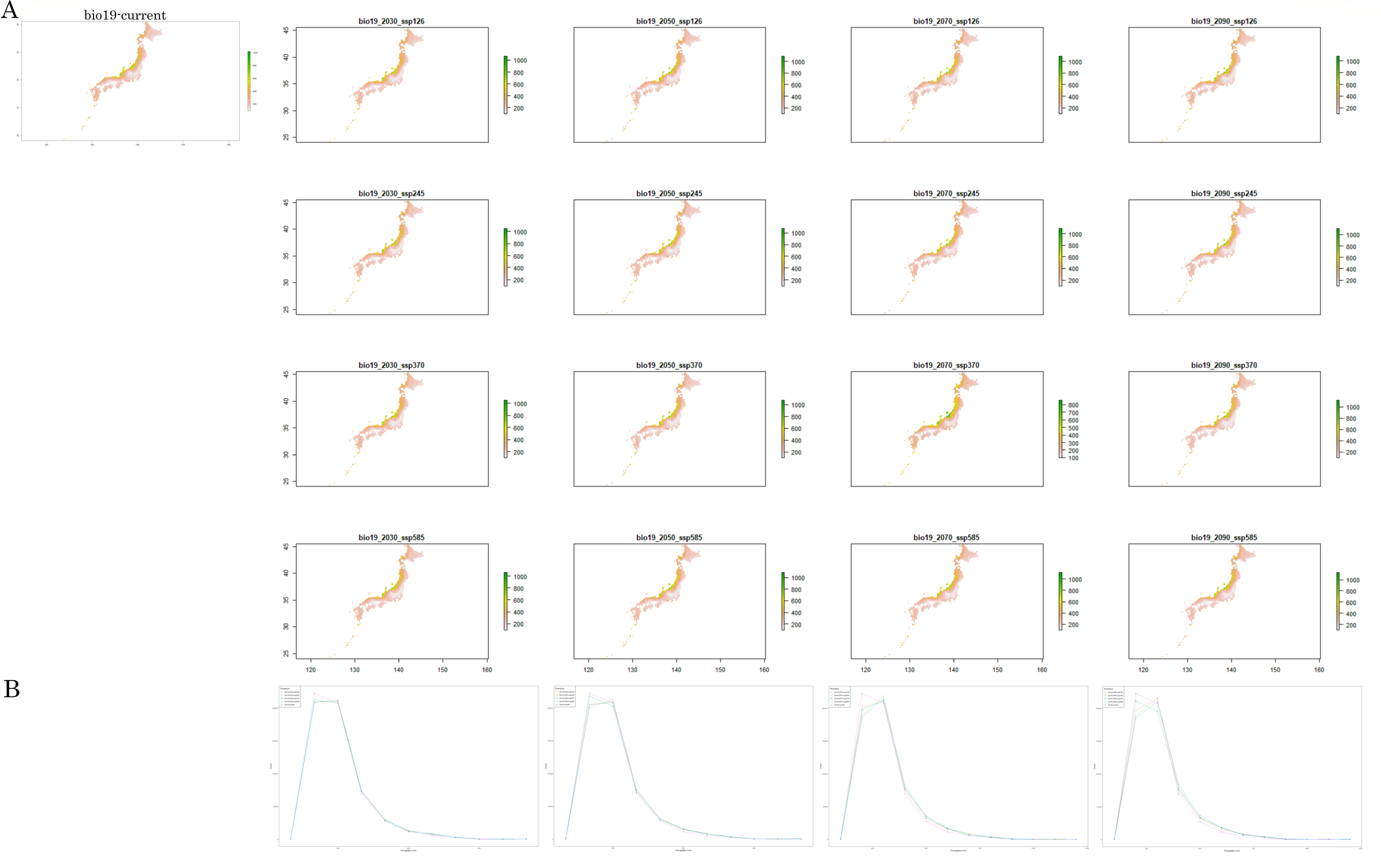

Supplement: S9 Fig — Spatial distribution (A) and counts (B) of each value for precipitation in the coldest quarter (Bio19) covariate. (TIFF) [file pone.0303137.s009.tiff]

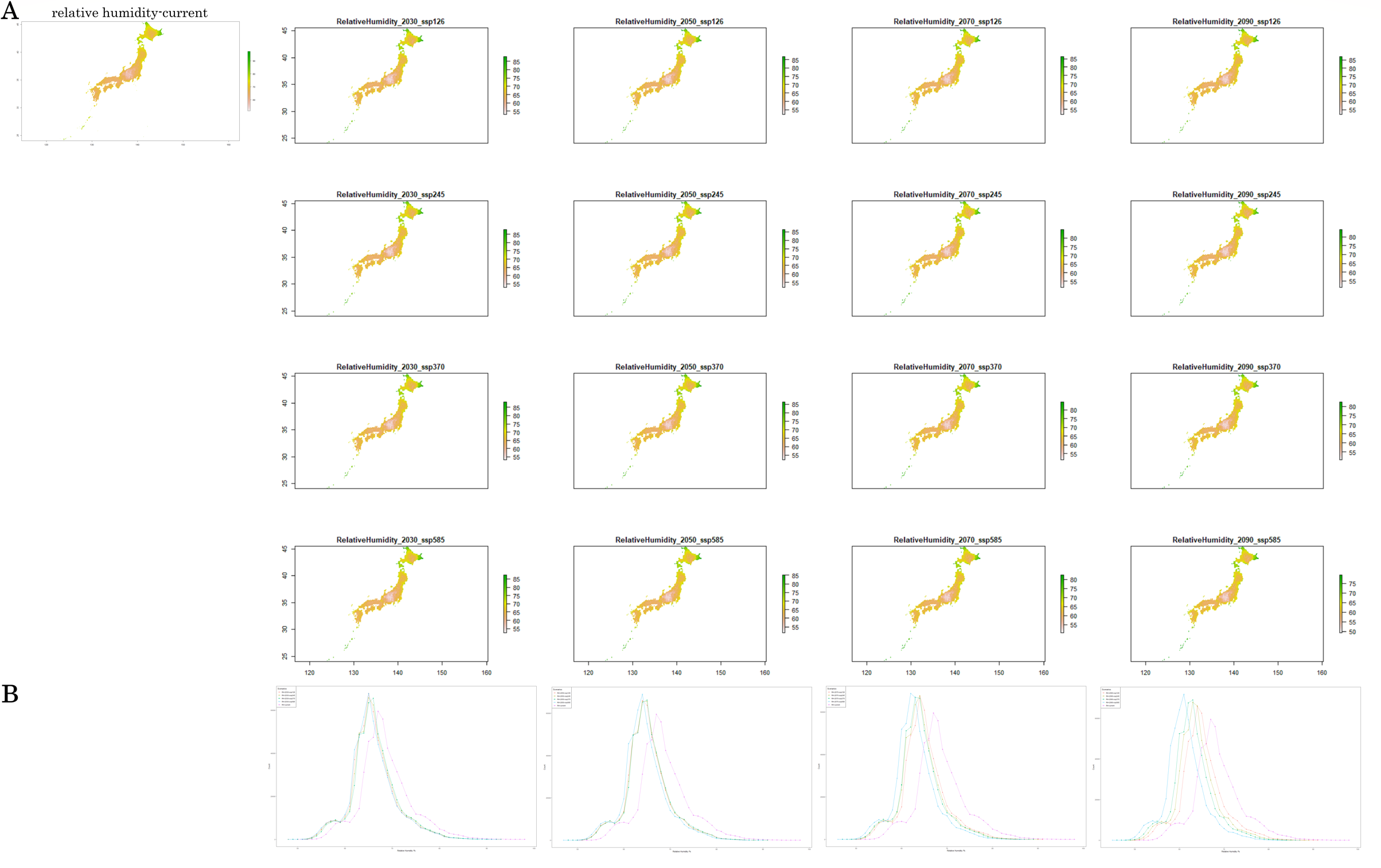

Supplement: S10 Fig — Spatial distribution (A) and counts (B) of each value for annual relative humidity (RH) covariate. (TIFF) [file pone.0303137.s010.tiff]

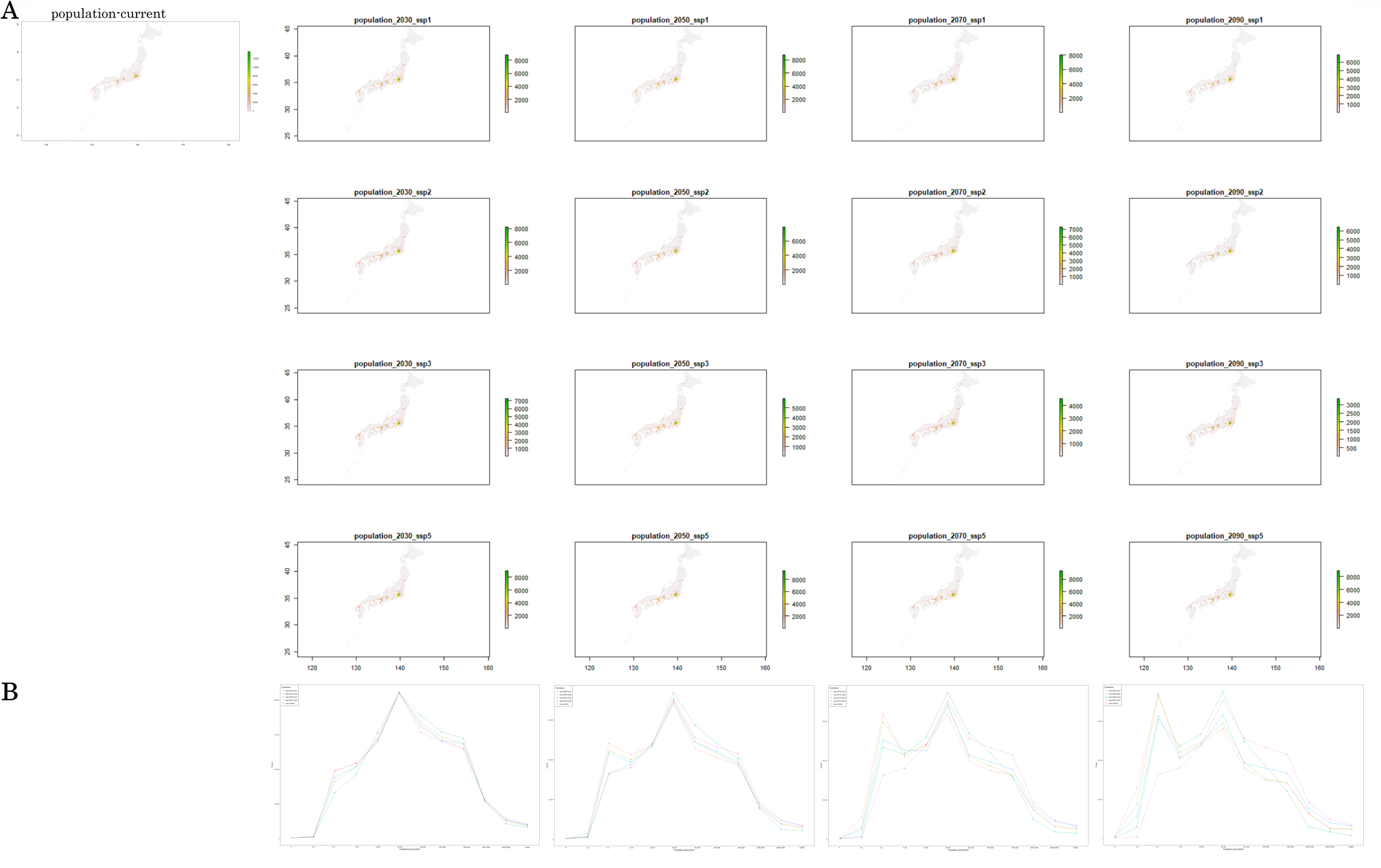

Supplement: S11 Fig — Spatial distribution (A) and counts (B) of each value for population (Pop) covariate. (TIFF) [file pone.0303137.s011.tiff]

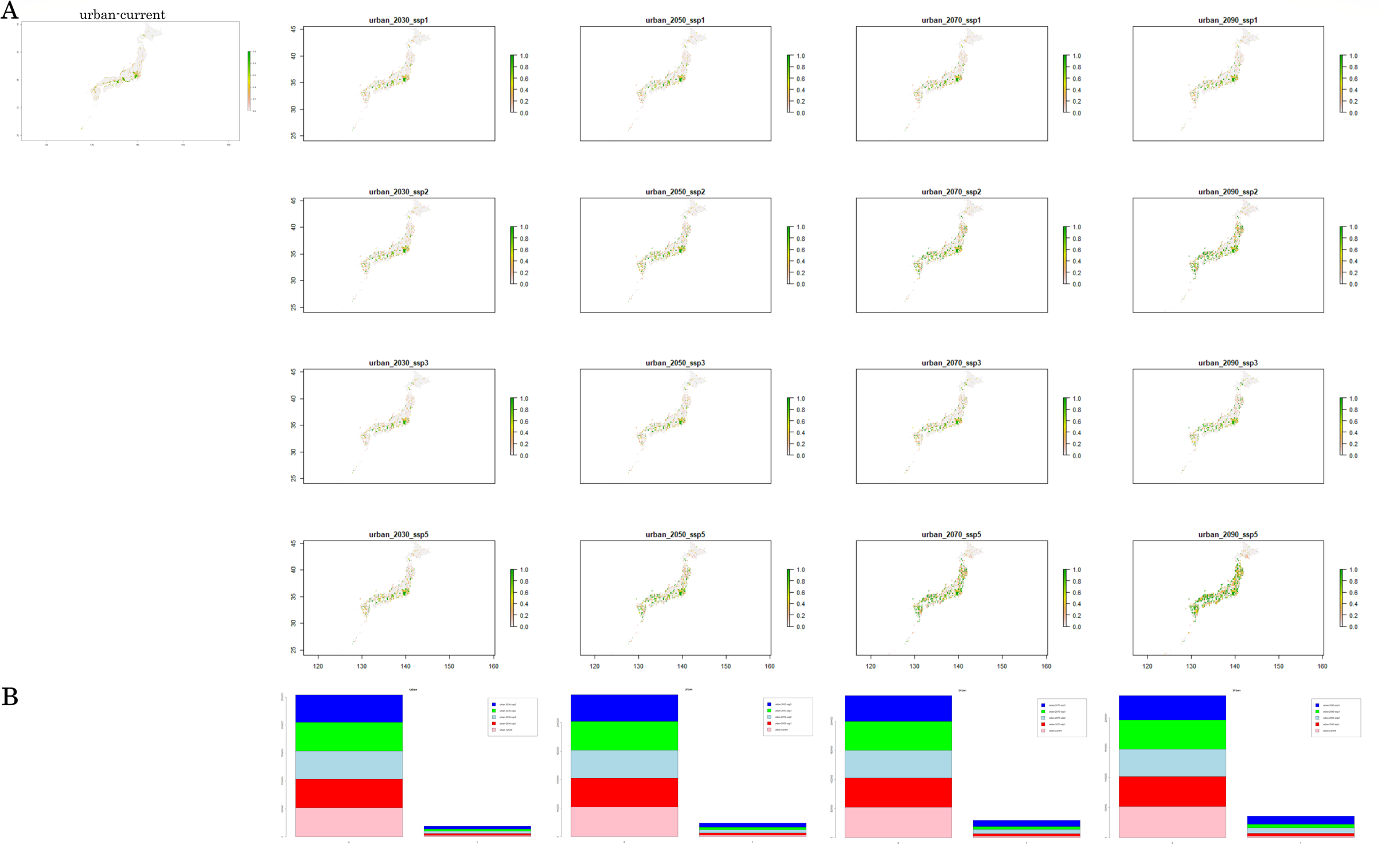

Supplement: S12 Fig — Spatial distribution (A) and counts (B) of each value for urban land fraction (Urban) covariate. (TIFF) [file pone.0303137.s012.tiff]

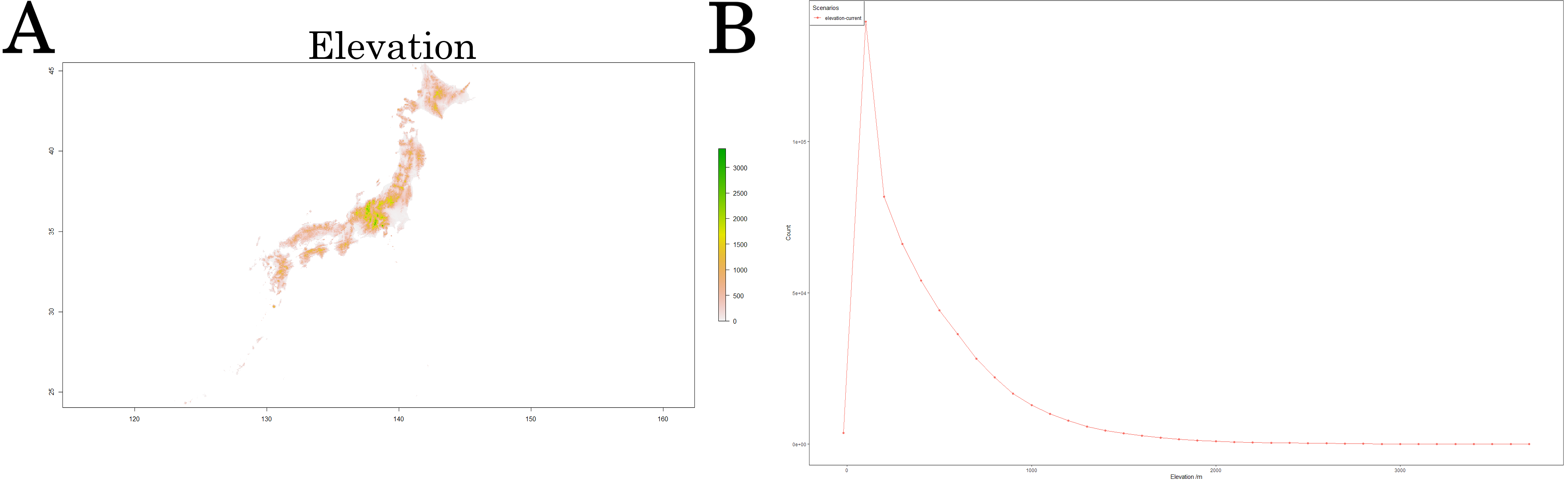

Supplement: S13 Fig — Spatial distribution (A) and counts (B) of each value for elevation (Elev) covariate. (TIFF) [file pone.0303137.s013.tiff]

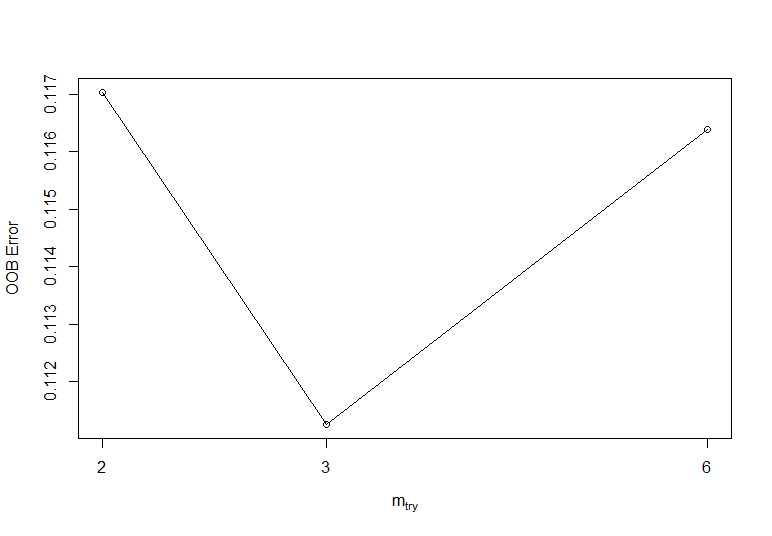

Supplement: S14 Fig — The lower the OOB error, the better the chosen number of variables. (TIFF) [file pone.0303137.s014.tiff]

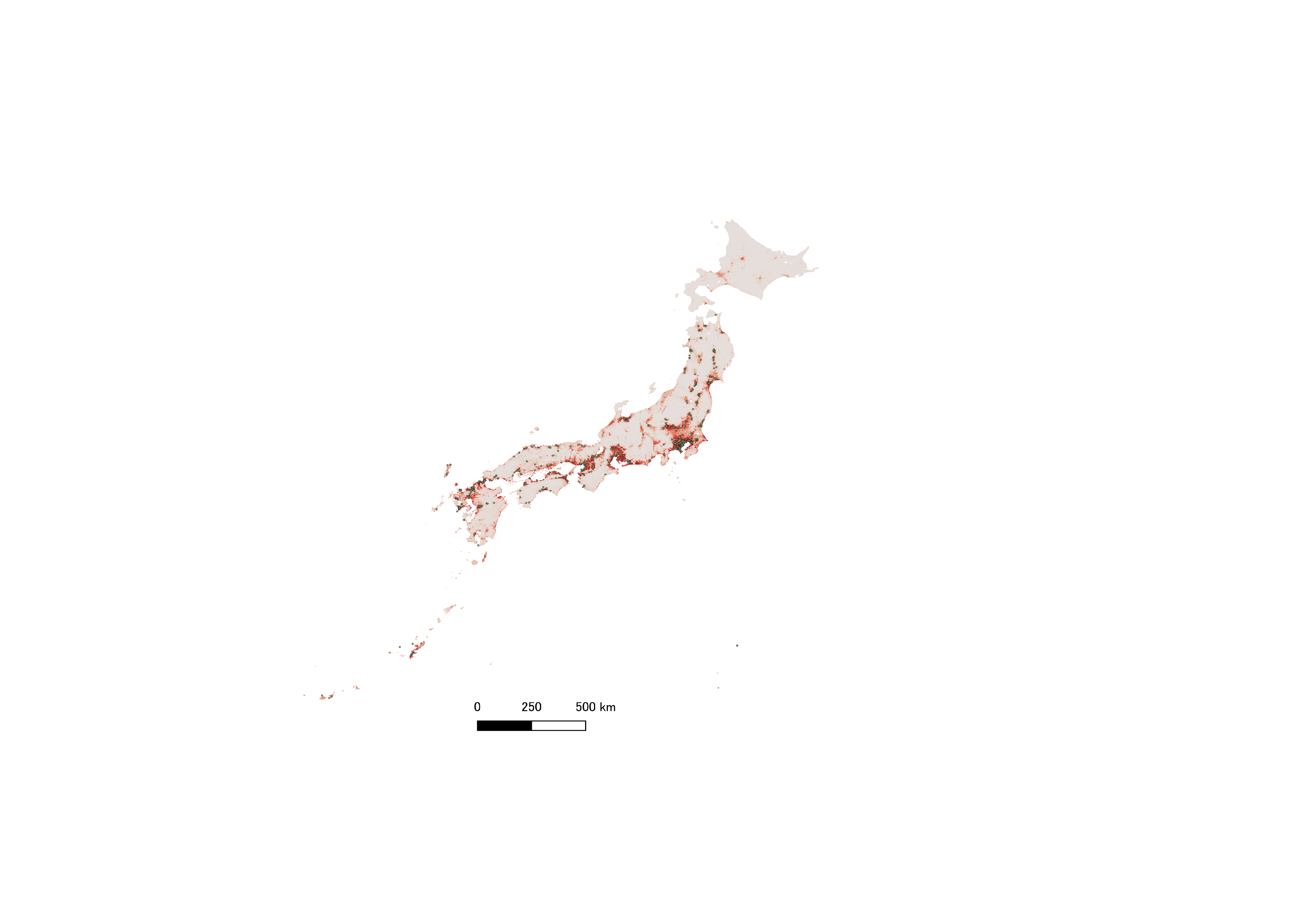

Supplement: S15 Fig — Blue dots represent presence data, and black dots represent (pseudo-)absence data. (TIFF) [file pone.0303137.s015.tiff]

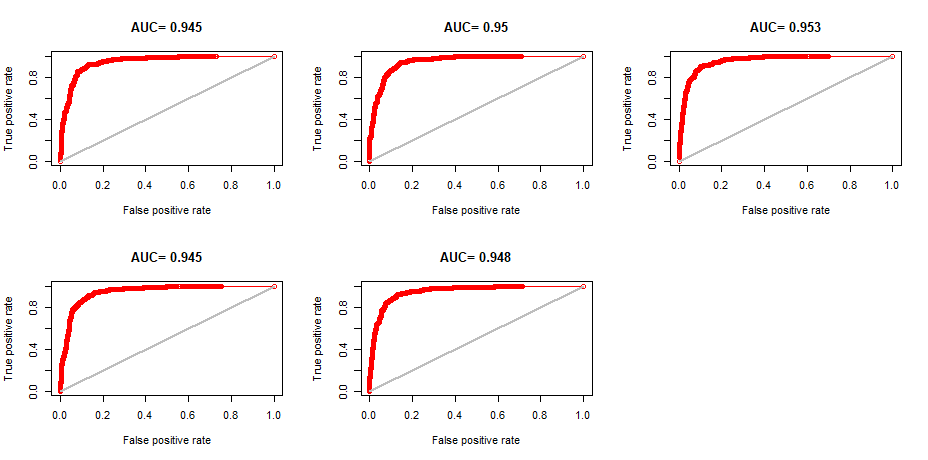

Supplement: S16 Fig — The higher the AUC value, the better the predictive performance. (TIFF) [file pone.0303137.s016.tiff]
